# Regulation of centriolar satellite integrity and its physiology

**DOI:** 10.1007/s00018-016-2315-x

**Published:** 2016-08-02

**Authors:** Akiko Hori, Takashi Toda

**Affiliations:** 1 Lincoln’s Inn Fields Laboratory, The Francis Crick Institute, 44 Lincoln’s Inn Fields, London, WC2A 3LY UK; 2 Developmental Biomedical Science, Graduate School of Biological Sciences, Nara Institute of Science and Technology (NAIST), 8916-5 Takayama, Ikoma, Nara 630-0192 Japan; 3 Department of Molecular Biotechnology, Hiroshima Research Center for Healthy Aging (HiHA), Graduate School of Advanced Sciences of Matter, Hiroshima University, 1-3-1 Kagamiyama, Higashi-Hiroshima, 739-8530 Japan

**Keywords:** Cellular stress, Centriole, Ciliogenesis, MSD1/SSX2IP, Microtubule, PCM1, PLK4, Phosphorylation, Ubiquitylation

## Abstract

Centriolar satellites comprise cytoplasmic granules that are located around the centrosome. Their molecular identification was first reported more than a quarter of a century ago. These particles are not static in the cell but instead constantly move around the centrosome. Over the last decade, significant advances in their molecular compositions and biological functions have been achieved due to comprehensive proteomics and genomics, super-resolution microscopy analyses and elegant genetic manipulations. Centriolar satellites play pivotal roles in centrosome assembly and primary cilium formation through the delivery of centriolar/centrosomal components from the cytoplasm to the centrosome. Their importance is further underscored by the fact that mutations in genes encoding satellite components and regulators lead to various human disorders such as ciliopathies. Moreover, the most recent findings highlight dynamic structural remodelling in response to internal and external cues and unexpected positive feedback control that is exerted from the centrosome for centriolar satellite integrity.

## Introduction

The centrosome plays multiple roles in many biological processes including cell proliferation, differentiation and development [1–4]. Since this structure was originally discovered by Edouard Van Beneden in 1883, and later named and further described by Theodor Boveri in 1888 [5–7], it has generally been accepted that most, if not all, of its cellular functions are executed through microtubule-organising activities; the centrosome is deemed to be a major microtubule-organising centre (MTOC) in animal somatic cells. Microtubules, dynamic hollow biopolymers composed of α-/β-tubulin heterodimers, are nucleated from the centrosome and thereby play diverse cellular roles in various processes including cell cycle progression, chromosome segregation, cell motility and polarisation, cell fate determination and ciliogenesis [8–14].

**Fig. 1:**
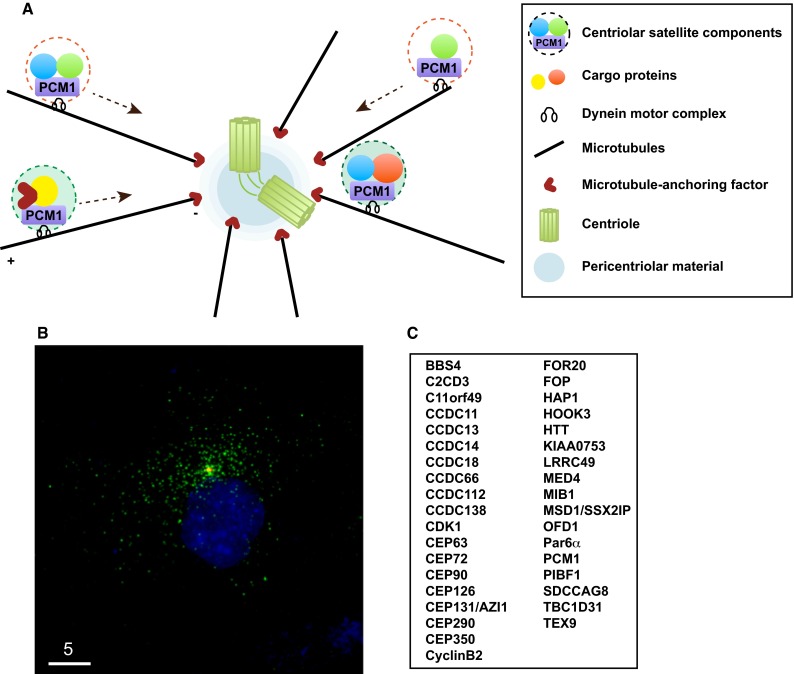
Centriolar satellites and their components. **a** Schematic presentation of the centrosome and centriolar satellites. PCM1 is a structural platform of centriolar satellites, which are localised along the microtubule and are moved around the centrosome by the dynein motor. **b** Immunofluorescence image. h-TERT-RPE1 cells were stained with anti-PCM1 (*green*) and γ-tubulin (*red*) antibodies. *Bar* 5 μm. **c** List of centriolar satellite components. This represents a very minimal set of proteins; a recent proteomic report [26] indicates that more proteins are localised to centriolar satellites

Classically, the centrosome is regarded as an organelle consisting of two structural sub-components, an orthogonally situated pair of centrioles and the surrounding proteinaceous substance called pericentriolar material (PCM) [15]. This definition has now been extended to a broader and more complex view as new structures designated centriolar satellites have emerged. Centriolar satellites are composed of numerous non-membrane particles (70–100 nm in size) found in the vicinity of the centrosome in mammalian cells. These structures were first recognised as electron dense masses around centrosomes as early as 1960s and then later as fibrous granules associated with basal body multiplication in differentiating multiciliated cells [16]. Subsequently, the major component comprising these structures was molecularly identified by the group of Tsukita and Shiina more than a quarter of a century ago; this was achieved through the characterisation of a protein called PCM1 [17] (Fig. 1a, b).

PCM1 was initially identified in human cells using human autoimmune antiserum [18], followed by cloning of its homologous gene from frog [17]. It is generally accepted that PCM1 comprises a structural platform for centriolar satellites; when PCM1 becomes dysfunctional, either by depletion, deletion or mutation, satellite particles disassemble. Therefore, the evaluation of new proteins as components of centriolar satellites is formally made according to the following two criteria: the first is colocalisation and physical interaction with PCM1, and the second is delocalisation from pericentrosomal locations upon PCM1 depletion [19–24].

A complete picture with regards to the full set of satellite components and the spatiotemporal constitution of centriolar satellites has not yet been established because the number of proteins identified as satellite components has continued to increase over the last several years. There were reported to be 11 in 2011 [16], ~30 in 2014 [25] and >100 according to the most recent studies [26–32] (Fig. 1c). The two main cellular functions of centriolar satellites, ciliogenesis and microtubule organisation, had already been posited by two earlier pioneering studies [17, 33]. Furthermore, genes encoding centriolar satellite components or regulatory proteins involved in centriolar satellite integrity have been identified as some that cause ciliopathy-related human diseases when mutated; these include Bardet–Biedl syndrome, Joubert syndrome, Meckel Gruber syndrome, primary microcephaly (MCPH) and oral-facial-digital syndrome [31, 34–41]. Molecular understanding of the cellular functions of centriolar satellites has increased, and further, broader roles than previously thought, such as those in autophagy and actin filament nucleation/organisation, have started to emerge [42–45]. We have been witnessing an exiting era in which not only the comprehensive structural constituents but also the physiologies of centriolar satellites have been uncovered. This review focuses on describing recent advances in the cellular regulation of centriolar satellite integrity and its physiological significances (please refer to excellent earlier reviews on centriolar satellite structures and functions) [16, 25].

## Intrinsic control of centriolar satellite integrity

### PCM1 as a structural platform

**Fig. 2:**
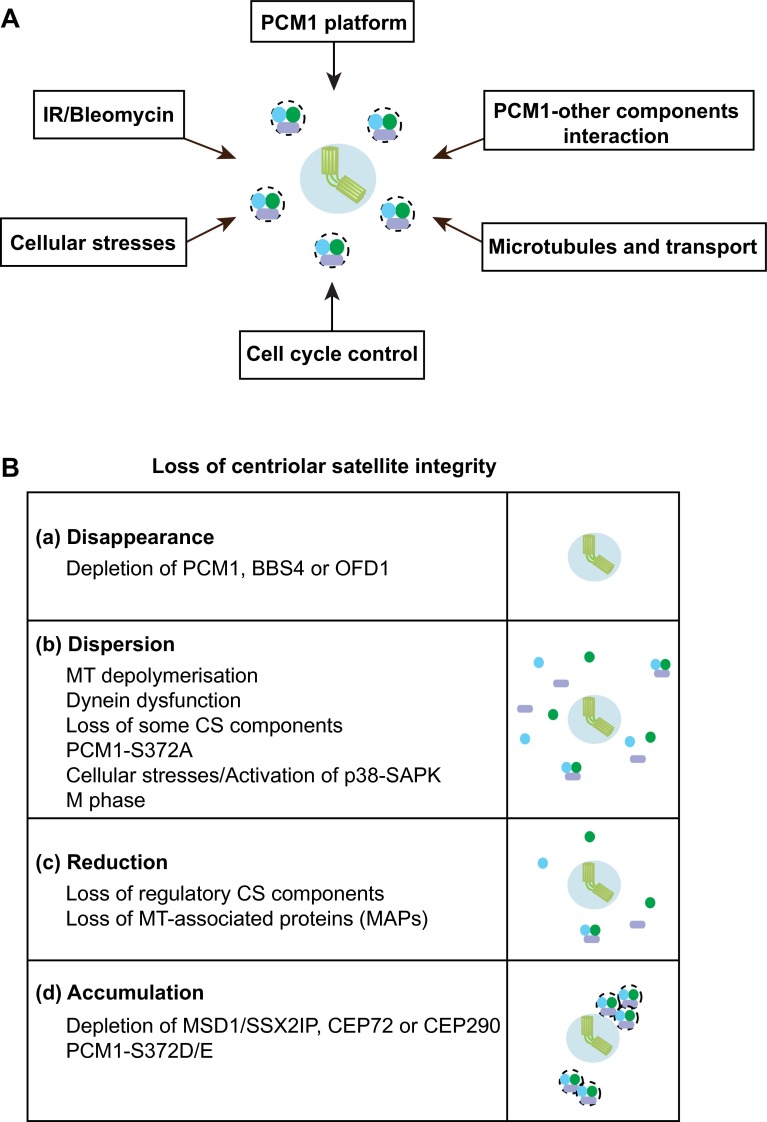
Factors and requirements that ensure centriolar satellite integrity. **a** Centriolar satellite organisation is regulated by a number of both intrinsic and external cues. **b** Outcomes of centriolar satellite integrity defects imposed by various conditions. *a* Disappearance. siRNA-mediated depletion of certain satellite components (e.g. PCM1, BBS4 and OFD1) leads to the disappearance of centriolar satellite particles. *b* Dispersion. Microtubule (MT) depolymerisation, impairment of the dynein motor, depletion of some components of centriolar satellites (CS), introduction of PCM1-S372A and exposure to various cellular stresses result in the dispersion of CS away from the centrosomal area. CS also becomes dispersed during M phase. Activation of the p38-SAPK MAP kinase pathway also compromises CS intensities. *c* Reduction. Depletion of regulatory satellite components leads to either the reduction of the number of CS particles or reduced intensities of CS. *d* Accumulation. Depletion of MSD1/SSX2IP, CEP72 or CEP290 or introduction of PCM1-S372D/E leads to abnormal accumulation of CS around the centrosome

PCM1 is a large protein (~230 kDa) rich in internal coiled coil domains [18] and play a scaffolding role in centriolar satellites as an assembly platform. This structural role of PCM1 is performed through self-oligomerisation and physical interaction with other components, mainly through its internal coiled coil domains. Consistent with this, PCM1 forms multimers in vitro and assembles into large aggregates in vivo when truncated proteins consisting of the internal coiled coil domains are produced [33, 46]. A subset of other components including BBS4 and OFD1 is required for the formation and maintenance of centriolar satellite particles [19, 35]. It is noteworthy, however, that, unlike PCM1, these two components also localise to the centrosome/centriole and the basal body/primary cilium [19, 20]. BBS4 plays a critical role in primary cilium biogenesis as a component of a multiprotein complex called the BBsome (the Bardet–Biedl syndrome protein complex) [47, 48], while OFD1 directly regulates centriole architecture and ciliogenesis [49]. This suggests that these two proteins play important, physiological roles in centrosome structure and function other than their roles as centriolar satellite subunits. This implies that PCM1 is a bona fide platform for centriolar satellites, in which other components play a regulatory role in centriolar satellite integrity (Fig. 2a, b).

### Interaction of PCM1 with other satellite components

In addition to BBS4 and OFD1, depletion of several other satellite components impairs the pericentriolar patterns of centriolar satellite localisation to varying degrees. These include CCDC11 [30], CCDC12 [26], CCDC13 [50], CCDC14 [23, 26], CCDC18 [26], CCDC66 [26], CEP63 [23, 51–53], CEP72 [54], CEP90 [55, 56], CEP126 [57], CEP131/AZI1 [26, 58–60], CEP290 (also called NPHP6) [38, 61–63], FOR20 [64], HAP1 and HTT [36, 65], KIAA0753 [23], Par6α [66] and SDCCAG8 [67]. However, in most of these cases, the underlying molecular mechanisms by which centriolar satellite integrity is disturbed have not yet been explored, and it remains to be determined whether alterations of centriolar satellite distributions are due to the failure of PCM1 oligomerisation, the compromised interaction of PCM1 with other components or perturbation of the association of PCM1 particles with microtubules (see below). It would be important to clarify whether these alterations of satellite patterns correspond to the disappearance, reduced numbers, lower intensities or spatial dispersion of centriolar satellite particles (Fig. 2b). Thus, how centriolar satellite integrity is intrinsically established is one of the critical issues to be addressed in the future.

## The microtubule cytoskeleton

### Association and transport

Centriolar satellites are localised along microtubules emanating from the centrosome. An intact microtubule network is essential to maintain centriolar satellite integrity because depolymerisation of microtubules by anti-microtubule agents such as Nocodazole or cold treatment results in the dispersion of satellite particles from the pericentriolar region towards the entire cytoplasm (Fig. 2a, b) [17, 33, 46]; consistent with this, knocking down a series of microtubule machinery (ANK2, DCTN1, MAPT, MAP7D1, MAP9, MAP7D3 and MAPRE3) affects intensities of centriolar satellites [26]. Satellite particles associated with microtubules are not static within a cell. Instead, particles rapidly and continuously move around the centrosome. The majority of particles appear to move towards the centrosome, and consequently these particles were originally termed satellites [17]. However, the precise manner of centriolar satellites movements in the proximity of centrosomes and the regulation of their steady state at this position are still unknown. Therefore, the dynamics of centriolar satellites in various conditions is a major question to be addressed in the field.

Despite this situation, it is shown that centrosome-oriented directional movement of centriolar satellites, at least in part, is driven by cytoplasmic dynein. The dynein complex physically interacts with satellite components [47], and the perturbation of dynein functions [68, 69] results in the dispersion of centriolar satellites [19, 33]. Depletion of two satellite components, CEP72 and CEP290, also results in the aggregation of centriolar satellites, which is attributed to the defective dissociation of centriolar satellites from dynein [20, 70] (Fig. 2b). Nonetheless, how PCM1 and hence centriolar satellites interact with the dynein complex remains to be established, although BBS4 [47] and Par6α [66] are reported to be required for the association between PCM1 and the dynein complex.

### Delivery of microtubule-anchoring and other regulatory factors to the centrosome

A large number of centriole/centrosome proteins are transported along with centriolar satellites as cargo molecules in a dynein-dependent manner [33]. The recently identified microtubule-anchoring factor MSD1/SSX2IP is one of these (see “Box for a detailed description of MSD1/SSX2IP”). In support of the significance of MSD1/SSX2IP localisation to centriolar satellites, depletion of PCM1, which disperses MSD1/SSX2IP from satellites, also results in microtubule-anchoring defects [24, 33]. Similar microtubule defects are also reported upon depletion of a series of satellite components that are required for proper localisation of PCM1 to the pericentriolar region; these include CEP90 [55, 56], FOR20 [64], HOOK3 [65] and Par6α [66]. Other proteins including CAP350, FOP, Ninein, ODF2/Cenexin1 and Trichoplein, among which FOP is a centriolar satellite component, are also involved in microtubule anchoring [33, 71–75]. However, it remains to be determined how these factors tether microtubules and whether these proteins physically/functionally interact with MSD1/SSX2IP. It is noteworthy that MSD1/SSX2IP may play a role in PCM1 localisation and centriolar satellite integrity independent of the microtubule anchoring; MSD1/SSX2IP could directly be involved in centriole satellite integrity.

Interestingly, in contrast to PCM1 depletion, which leads to the dispersion/disappearance of centriolar satellites [19, 33], knockdown of MSD1/SSX2IP renders the microtubule network present yet completely disorganised and importantly gives rise to the abnormal aggregation of larger satellite particles in the vicinity of the centrosome (Fig. 2b). Furthermore, these satellite particles are stuck at the minus ends of disorganised microtubules with greatly reduced motility [24, 26, 28].

### Centriole assembly and centrosome copy number

Intriguingly, upon MSD1/SSX2IP depletion in U2OS and many other cancer cells, satellite aggregates contain, besides constitutive satellite components, a number of structural constituents of centrioles/centrosomes that are not normally localised to centriolar satellites. These include centrin (normally localises to the distal lumen of centrioles) [76, 77], centrobin (the daughter centriole) [78], CEP164 (the distal appendages) [79], C-NAP1 (the proximal end) [80] and CP110 (the distal end) [81]. It is of note that centriolar satellites are not responsible for transport of all the centrosome/centriole components. For instance, PLK4 (the proximal end) [82], CEP152 (the proximal end) [83, 84], hSAS-6 (procentriole) [85], pericentrin (PCM) [86] and γ-tubulin (the PCM) [87] do not accumulate as satellite aggregates upon depletion of MSD1/SSX2IP [28].

Electron and super-resolution fluorescence microscopy analyses revealed that depletion of MSD1/SSX2IP leads to compromised centriole morphologies. Under this condition, cylindrical centriole structures tend to be lost and centriolar proteins that accumulate within satellite aggregates are not localised to the normal centriolar position [28]. These results are perfectly in line with earlier studies that showed centriolar satellites are important for proper centrosome/basal body assembly [19, 20, 33, 88]. Consistent with compromised centriole assembly, MSD1/SSX2IP-depleted cells are insensitive to PLK4 overproduction, which otherwise induces centriole/centrosome overamplification using a pre-existing centriole as a template [82, 89–91].

Remarkably, U2OS cells in which MSD1/SSX2IP is depleted exhibit accelerated centrosome reduplication upon hydroxyurea (HU)-mediated arrest [92, 93], which might look like the opposing impact exerted by MSD1/SSX2IP depletion under PLK4 overproduction described earlier. One possible scenario is that because several centriolar components prematurely accumulate in satellites upon MSD1/SSX2IP depletion, these cells are likely to undergo faster and more efficient overamplification of centrosomes. Similar, if not identical, accumulation of satellite aggregates (supernumerary centriole-related structures) is reported upon depletion of CEP131/AZI1 [59] and CCDC14 [23], suggesting that these two proteins are involved in transport of centrosomal/centriolar proteins from the cytoplasm to the centrosome/centriole and may functionally interact with MSD1/SSX2IP.

Centriolar satellites ensure centriole duplication by helping assembly of a series of centriolar/centrosomal components including CEP63, CDK5RAP2/CEP219 [94, 95], CEP152 and WDR62 [96, 97], thereby securing centrosome recruitment of CDK2 [31], a critical regulator of centrosome duplication [98, 99]. Intriguingly, these assembled centriolar/centrosomal components are collectively termed MCPH-associated proteins because mutations in the corresponding genes are identified in human patients suffering from MCPH [100, 101]. These results highlight the complex, multi-layered roles of centriolar satellites; centriolar satellite integrity plays both positive and negative roles in centriole/centrosome assembly and duplication. A similar dual role of centriolar satellites in ciliogenesis was also reported [102]. This raises the intriguing possibility that each particle of centriolar satellites may not be equal in its composition, but instead they may be composed of various combinations of satellite components and regulators, which serve a diverse set of functions.

**Fig. 3:**
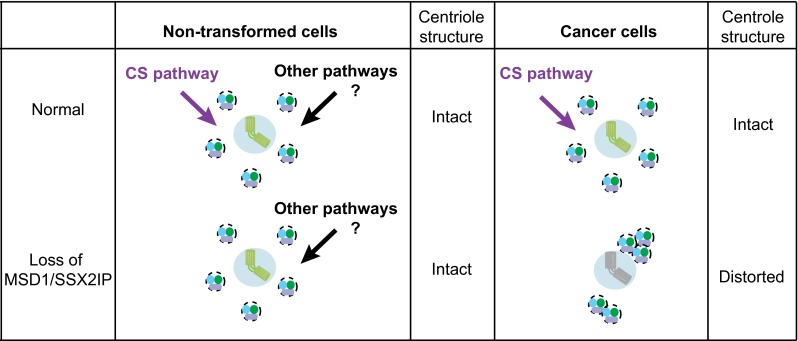
Multiple pathways ensure centrosome assembly, which is compromised in cancer cells. In non-transformed cells (*left*), centriolar assembly is apparently normal upon depletion of MSD1/SSX2IP, although the microtubule network is disorganised. Alternate compensatory pathways that ensure building of the proper centriole structure may be exploited in these cells. On the other hand, the absence of MSD1/SSX2IP leads to alteration of centriolar satellite organisation in many cancer cells (*right*); centriolar satellites carry centriolar/centrosomal components and are stuck at microtubule minus ends in the vicinity of the centrosome, leading to faulty assembly of centrioles

Of note, abnormal accumulation of satellite particles in the absence of microtubule anchoring (i.e. MSD1/SSX2IP depletion) is observed only in cancer-derived culture cells (U2OS, HeLa, MCF-7, A549, T98G and Saos-2); non-transformed cells (RPE1, W138 and MG00024B) do not display satellite aggregation [28]. This implies that normal cells are equipped with additional systems besides centriolar satellites by which to secure centriole/centrosome assembly (Fig. 3). Consistent with this proposition, a number of recent studies indicate the existence of several centriolar satellite-independent pathways. These include a CEP76-CEP290-CP110-dependent pathway [103], an LGALS3BP-mediated signalling pathway [104], a Rab11 and endosome pathway [105], a ninein–centriolin–CDK5RAP2/CEP215–pericentrin-mediated delivery pathway [73, 86, 95, 106, 107] and a CEP63-CCDC14-KIAA0753-mediated centriole assembly pathway [23, 108]. How these multiple pathways interact and cooperate to form a functional network for proper centriole/centrosome assembly remains to be determined, and this is the future research direction in this field.

## Cell cycle-dependent regulation of centriolar satellite organisation

The cellular localisation of centriolar satellites exhibits dynamic behaviour during the cell cycle. In the very first study of human PCM1 [18], the authors found that the centrosomal signals detected with anti-PCM1 antibodies were reduced during G2 phase, remained low in M phase and then increased towards the following G1 phase, although the antibodies used seemed to fail to recognise the pericentrosomal localisation of PCM1. The notion that centriolar satellites completely disassemble and disappear from pericentrosomal regions during M phase varies among subsequently published studies; some detected mitotic satellite signals of PCM1 [74], while others claimed that these signals exhibited reduced intensities or even disappeared [16, 19, 24, 46]. It would be fair to describe that their mitotic intensities at the pericentrosomal region are more or less decreased during mitosis (Fig. 2a), although this does not imply that centriolar satellites do not play any physiological roles in M phase. In fact, PCM1 reportedly is involved in spindle pole integrity during metaphase [74]. How this spatial regulation of centriolar satellites materialises at the molecular level remains elusive, and this area has been poorly characterised.

Another interesting issue with regards to cell cycle-dependent regulation of centriolar satellite integrity arises when cells exit from mitosis and initiate ciliogenesis. Under this condition, depletion of TALPID3 and/or CEP290 leads to an aberrant distribution of centriolar satellites [109]. TALPID3 is known to be localised to the extreme distal end of centrioles and required at least during ciliogenesis for proper localisation of Rab8a, a key small GTPase involved in protein trafficking. It would be of great interest to explore how TALPID3, CEP290 and Rab8a regulate centriolar satellite integrity outside the mitotic cycle.

## Cellular stress responses and centriolar satellite integrity

### Remodelling of centriolar satellites upon cellular stress through the p38-SAPK signalling pathway

**Fig. 4:**
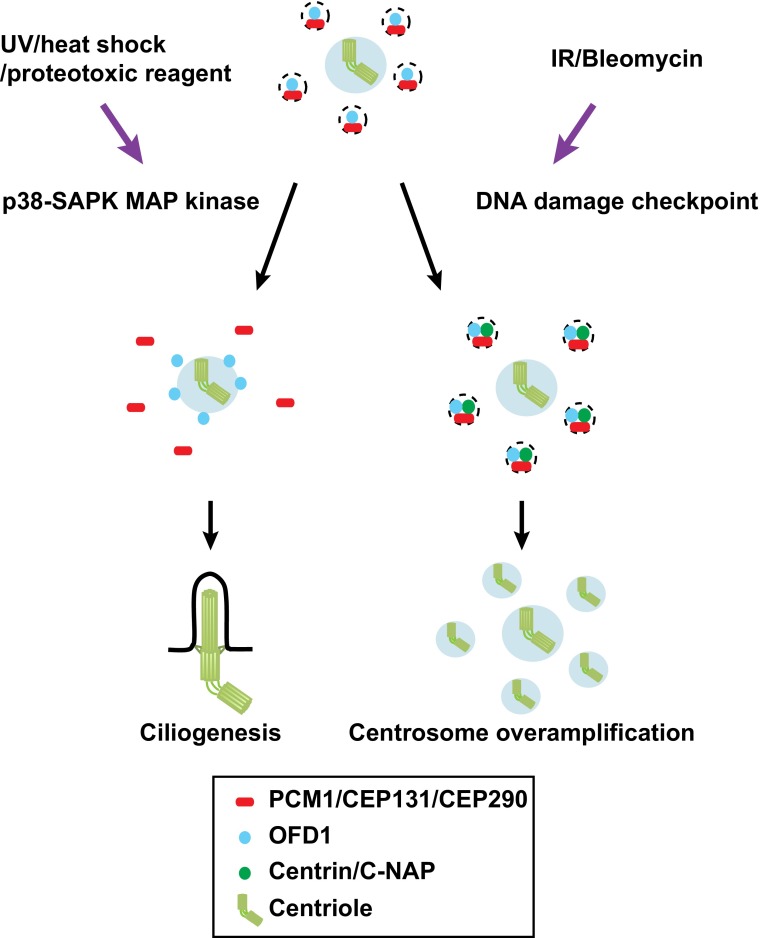
Centriolar satellite remodelling upon exposure to various cellular stresses. Several cellular stresses including exposure to UV light, heat shock and proteotoxic reagents activate the p38-SAPK MAP pathway, thereby inducing the dissociation of PCM1. CEP131/AZI1 and CEP290 (*red*) form centriolar satellite particles, while OFD1 (*blue*) is retained as a satellite component (*left*). By contrast, exposure to IR or Bleomycin activates the CHK1-dependent DNA damage checkpoint pathway, resulting in overamplification of centrioles, in which centriolar satellites are used as intermediate precursors of supernumerary centrioles (*right*)

Cells are constantly exposed to both external and internal stresses and damage, and consequently they have developed numerous protective strategies by which to tackle these adverse challenges. Recent studies indicate that centriolar satellites are also under the control of such stress responses. A variety of stresses including exposure to ultraviolet (UV) light, heat shock and proteotoxic reagents lead to remodelling of centriolar satellites; a number of satellite components including PCM1, CEP131/AZI1 CEP290 and MSD1/SSX2IP become acutely dispersed from the pericentriolar region (Fig. 2a, b) [58]. Interestingly, under this condition, ciliogenesis is promoted (Fig. 4). Cellular stresses activate the stress-activated p38-SAPK MAP kinase pathway [110]. In fact, satellite remodelling upon stresses requires p38; activated p38 leads to enhanced interaction between PCM1 and CEP131/AZI1 in the cytoplasm away from the pericentriolar region, where these two proteins are normally localised. p38 activates the downstream kinase MK2, which in turn phosphorylates CEP131/AZI1, thereby creating a binding pocket for the phospho-adaptor 14-3-3 [111]. 14-3-3-associated CEP131/AZI1 binds tightly to PCM1, which results in the blockage of new satellite formation (Fig. 4).

In addition to satellite remodelling, cellular stresses inhibit MIB1, an E3 ubiquitin ligase enzyme that is also a component of centriolar satellites. Interestingly, MIB1 inhibition is independent of p38 activation [58]. Thus, it appears that exposure to cellular stresses impacts on two bifurcated downstream branches; one is p38-mediated centriolar satellite remodelling, while the other is p38-independent inhibition of MIB1 that ubiquitylates PCM1, CEP131/AZI1 and CEP290. Neither MIB1 nor MIB1-mediated ubiquitylation of PCM1 and CEP131/AZI1 plays any roles in the association of CEP131/AZI1 and PCM1 with centriolar satellites, indicating that MIB1 is not involved in overall centriolar satellite integrity [58]. Under non-stressed conditions, MIB1 actively ubiquitylates PCM1, CEP131/AZ1 and CEP290, which suppresses the interaction between CEP131/AZI1 and PCM1 and simultaneously inhibits ciliogenesis. Upon exposure to stresses, satellite remodelling occurs, leading to promotion of cilia formation [58].

More recent work shows that PCM1 in turn is essential for tethering MIB1 to centriolar satellites. In *PCM1* knockout human cells, MIB1 becomes localised to the centrosome, thereby destabilising TALPID3 through poly-ubiquitylation. The consequent reduction of TALPID3 leads to abrogation of recruitment of ciliary vesicles, resulting in suppression of cilium assembly [112]. It would be worth pointing out that whether the MIB1 ubiquitin ligase catalyses mono-ubiquitylation [58] or poly-ubiquitylation (and destabilisation) of PCM1 and CEP131/AZI1 [112] remains to be solved. In addition to centriolar satellite components (CEP131/AZI1, CEP290 and PCM1), MIB1 ubiquitylates PLK4, which leads to degradation of this protein [32]. This role of MIB1 in PLK4 stability may at least in part account for the negative role of this ubiquitin ligase in ciliogenesis as well as MIB1-mediated destabilisation of TALPID3 [32, 112]. Admittedly a complicated network is in action to regulate centriolar satellite integrity through the MIB1-ubiquitin pathway and its substrates.

It is of interest to point out that satellite-localising PCM1 is generally believed to be important to promote ciliogenesis [19, 20, 109, 113]. The result described earlier clearly indicates a more complex mode of centriolar satellite functions in controlling ciliogenesis because dispersed PCM1 (forming a complex with CEP131/AZI1) is still able to promote or even more potently induce ciliogenesis [58]. It should also be noted that, under stress conditions, centriolar satellites (detected by OFD1) are devoid of PCM1, CEP131/AZI1 and CEP290, which become dispersed. This implies that, as mentioned earlier, centriolar satellites comprise multiple particles with distinct constituents and that even PCM1-independent centriolar satellites may exist upon exposure to certain cellular stresses [58]. Unlike the prevailing view that PCM1 is the main structural platform for centriolar satellites, the composition and organisation of centriolar satellites would be rewired within various environmental, developmental and cell cycle contexts. More work including electron microscopy and proteomics is necessary to further explore these intriguing findings.

### DNA damage-induced centrosome overamplification and the DNA damage checkpoint

DNA damage imposed by ionising radiation (IR) or chemicals such as Bleomycin induces centrosome amplification via formation of excessive centriolar satellites (Figs. 2a, b, 4) [114]. The DNA damage checkpoint signalling pathway mediated by CHK1 is activated under this condition and is essential for this response. An earlier report regarding the role of centriolar satellites as intermediate precursors for centrosome amplification is consistent with this finding [115]. Prolonged G2 arrest leads to centrosome overamplification [116]. Therefore, it is likely that IR or Bleomycin activates the DNA damage checkpoint, which leads to G2 delay, thereby inducing centrosome overamplification.

It is worth noting that DNA damage appears to give rise to two different, apparently opposing effects on centriolar satellite integrity. On one hand, it results in the remodelling of centriolar satellites, in which PCM1 as well as CEP131/AZI1 and CEP290 becomes dispersed away from the pericentriolar region, and yet promotes ciliogenesis [58, 111]. On the other hand, it leads to centrosome overproduction using centriolar satellites as intermediate precursors (Fig. 4) [114]. To reconcile these results, we point out a few differences in experimental procedures between the two situations. One lies in the DNA-damaging methods and downstream pathways that are activated. The former studies [58, 111] used UV treatment, which activates the p38-SAPK pathway, while the latter study [114] used IR and Bleomycin, which activate the CHK1-DNA damage checkpoint pathway. The second difference is the experimental timeline. In the former reports [58, 111], acute responses were observed (1 h after UV treatment), while in the latter report [114], cells were observed at much later time points (24–72 h after irradiation). In any case, collectively, these findings uncovered the hitherto unknown spatiotemporal dynamics of centriolar satellite organisation upon exposure to various types of cellular stresses.

## An unexpected role of PLK4 in centriolar satellite integrity and ciliogenesis

### Phosphorylation of PCM1 by PLK4

As described earlier, centriolar satellites play a critical role in centriole/centrosome assembly and ciliogenesis. Is there any converse regulation? A recent study showed that this is indeed the case [117]. PLK4 is regarded as a rate-limiting master regulator of centriole copy number control because centrioles fail to duplicate when PLK4 malfunctions but conversely undergo overamplification when PLK4 is overproduced [82, 118–120]. PLK4 plays an essential role during mouse development [121, 122]. Furthermore, the importance of PLK4 for proper cell proliferation and differentiation in humans is underpinned by recent studies demonstrating that mutations in *PLK4* lead to primordial dwarfism and that abnormal gene amplification results in human embryos exhibiting aneuploidy [123–125]. Several in vivo substrates in addition to PLK4 itself [126] have been identified that are localised to the centriole/centrosome and play an important role in centriole duplication. These include STIL/hSAS-5 [127–129], FBXW5 (a component of the SCF^FBXW5^ ubiquitin ligase) [130] and GCP6 (a component of the γ-tubulin complex, γ-TuC) [131, 132]. These results led to the consensus in the field that PLK4 exerts its critical role in centriole duplication through phosphorylating centriole/centrosome components [123, 133].

**Fig. 5:**
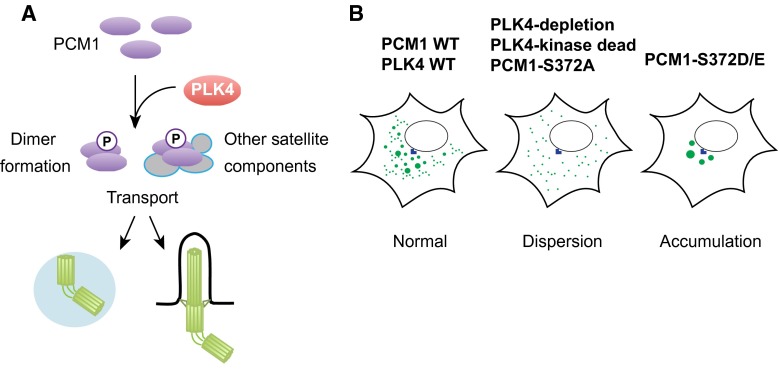
Phosphoregulation of PCM1 through PLK4. **a** PCM1 is phosphorylated through PLK4. PLK4 phosphorylates the conserved S372 residue within PCM1. This phosphorylation plays a critical role in PCM1–PCM1 dimerisation and interaction with other centriolar satellite components. Phosphorylated PCM1 is responsible for transport of a number of proteins that are important for assembly of the centriole/centrosome/basal body. **b** Impact of PLK4 and PLK4-mediated phosphorylation of PCM1 on centriolar satellite integrity. Under normal conditions, centriolar satellites are localised to the pericentriolar region (*left*). Upon depletion of PLK4 or introduction of kinase-dead PLK4 or non-phosphorylatable PCM1 (PCM1-S327A), satellite particles become dispersed (*middle*). By contrast, introduction of phosphomimetic PCM1 (PCM1-S372D/E) leads to abnormal accumulation of satellite particles around the centrosome (*right*)

Surprisingly, PLK4 depletion or introduction of kinase-dead PLK4 to PLK4-depleted cells leads to the dispersion of centriolar satellite particles (PCM1, CEP290 and MSD1/SSX2IP) from the pericentriolar region [117]. Importantly, this phenotype arises much earlier when the normal number of centrioles is still retained, and is observed even in G1-arrested cells when centrioles do not duplicate, indicating that satellite dispersion and centriolar duplication deficiency are separate and independent outcomes. Consistent with this notion, depletion of hSAS-6, a critical factor for centriole duplication [85], does not lead to satellite dispersion [117]. PCM1 was shown to be a phospho-protein and to interact with PLK4 by a proximity-dependent biotin identification method [23, 134–136]. Semi-quantitative mass spectrometry analysis identified S372 as a phosphorylation site that is dependent upon PLK4. S372 is conserved across a wide variety of eukaryotes. Because PLK4 binds to phosphorylated PCM1 in vivo, and PLK4 and PCM1 directly interact in vitro [117], PCM1 is likely an in vivo substrate of PLK4 (Fig. 5a).

Introduction of the non-phosphorylatable PCM1 mutant (PCM1-S372A) recapitulates the phenotypes of PLK4-depleted and kinase-dead PLK4-expressing cells. Conversely, the phosphomimetic mutant (PCM1-S372D/E) rescues the dispersed centriolar satellite patterns (Figs. 2b, 5b); however, the suppression is only partial and under this condition centriolar satellites are localised only around the centrosome in a more concentrated, non-motile manner. Importantly, PCM1 phosphorylation is required to promote ciliogenesis, which is independent of centriole duplication [117].

What are the molecular consequences of PCM1 phosphorylation in terms of the maintenance of centriolar satellite integrity? Two functions of PCM1 crucial for ensuring centriolar satellite organisation are regulated by PLK4-mediated phosphorylation. The first is PCM1 self-dimerisation and the second is the interaction of PCM1 with BBS4 and CEP290, two components of centriolar satellites. In line with these results, S372 is located in the region of the second coiled domain of PCM1, which is important for protein–protein interactions [25]. Cumulatively, these findings revise our current view with regards to PLK4 functions and centriole satellite integrity; PLK4 plays a decisive role in centriole duplication by phosphorylating not only centriole/centrosome components, but also PCM1, which in turn secures centriole/centrosome assembly.

As described earlier, centriolar satellites disassemble during mitosis and reassemble in the following G1 phase [19, 33]. Given that PCM1 is phosphorylated not only by PLK4 but also by CDK1 and PLK1 [136, 137], it is tempting to speculate that CDK1- and/or PLK1-dependent phosphorylation promotes satellite disassembly during M phase, followed by PLK4-mediated phosphorylation of S372 during G1 phase, which promotes reassembly of centriolar satellites. This dual phospho-regulation or PCM1 may underlie at least in part the temporal regulation of cell cycle-dependent satellite organisation and remodelling. It is worth noting that it was recently shown that CDK1 and PLK4 play antagonistic roles in centriole duplication, negative and positive, respectively, through phosphorylation of STIL in a cell cycle-dependent manner [129].

### Human diseases

It is generally believed that ciliogenesis defects observed in cells in which PLK4 is depleted by siRNA, inactivated by a small molecule inhibitor or mutated are attributed to centriole duplication defects [123, 133]. However, under these conditions, centrioles and basal bodies still exist [123, 138]. It is, therefore, possible that the complete disappearance of the centriole/basal body is not an absolute prerequisite for ciliogenesis defects derived from PLK4 dysfunction. A higher level of PLK4 activity may be required for centriolar satellite integrity than for centriole duplication. PLK4 likely potentiates ciliogenesis to some degree through PCM1 phosphorylation. This safeguards centriolar satellite integrity, promotes delivery of ciliary components to the basal body and helps to build these structures. Therefore, PLK4 inactivation may induce anomalies in humans to some extent through centriolar satellite dispersion leading to ciliogenesis failure; this may account for the underlying aetiology of defective cilia diagnosed in human diseases caused by *PLK4* mutations.

## Autophagy and centriolar satellite integrity

An unexpected functional link was recently uncovered between autophagy and centriolar satellite integrity [42, 43, 139]. In proliferating cells in nutrient-rich conditions, basal autophagy inhibits ciliogenesis by degrading IFT20, an essential protein for primary cilium formation [43]. Under this condition, OFD1 locating at centriolar satellites is a critical player in this inhibition [42]. By contrast, upon serum deprivation, which induces both ciliogenesis and autophagy, induced autophagy actively degrades OFD1, thereby promoting ciliogenesis.

A positive role of PCM1, a platform for centriolar satellites, in ciliogenesis is well established [19, 20, 109, 112]. This positive function is executed through satellite- and microtubule-mediated transport of proteins required for basal body assembly and primary cilium biogenesis [24]. Thus, centriolar satellites play both positive and negative roles in ciliogenesis. As mentioned earlier, various cellular stresses promote ciliogenesis through satellite remodelling, in which OFD1 forms non-canonical centriolar satellites without PCM1 [58, 111]. Given the inhibitory role of OFD1 in ciliogenesis, OFD1-containing satellites assembled under acute stress conditions may represent the inactive form of OFD1 (e.g. the sequestration of OFD1 as abortive aggregates), thereby eliminating its inhibitory impact on ciliogenesis. It would be of great interest to dissect the comprehensive molecular composition of centriolar satellites under serum starvation conditions, to decipher to what extent centriole satellites are remodelled and to finally explore the mechanism by which satellite remodelling is induced.

## Neurogenesis and PCM1

One recent report [140] shows that a microRNA, called miR-128, negatively regulates the cellular levels of PCM1. Overexpression of miR-128 resulted in downregulation of PCM1, leading to repressing proliferation of neural progenitor cells yet simultaneously promoting their differentiation into neurons. Conversely, the reduction of miR-128 elicited the opposite effects; promotion of proliferation and suppression of differentiation of neural progenitor cells [140]. Whether this inhibitory role of PCM1 in neurogenesis is exerted through centriolar satellite integrity is yet to be rigorously examined, but this would be a novel, intriguing aspect of the physiology of centriolar satellites.

## Concluding remarks

**Fig. 6:**
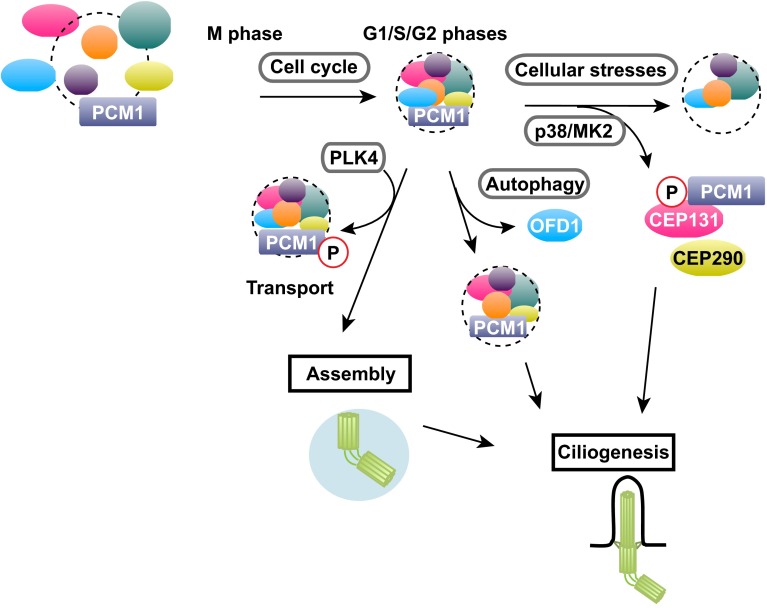
Diverse forms of centriolar satellites in the context of various cues and cell cycle stages. Centriolar satellite integrity is regulated by many factors. Centriolar satellite organisation is changed during the cell cycle. Centriolar satellite integrity is structurally and functionally linked to other cellular processes including autophagy. In addition, centriole satellites undergo drastic remodelling in response to various cellular stresses. A cohort of protein kinases including PLK4 and p38-MK2 are involved in centriolar satellite integrity, which plays a pivotal role in centriole/centrosome assembly and ciliogenesis. See text for details

Since their discovery a quarter of a century ago, our understanding of the components and functions of centriolar satellites has progressed rapidly, particularly during the last decade. These structures play critical roles in centriole/centrosome assembly, ciliogenesis and autophagy. A number of factors, both intrinsic and external cues, regulate centriolar satellite integrity. Recent studies have started to uncover the dynamic regulation of centriolar satellite organisation and indicate that centriolar satellites are composed of multiple forms of individual particles that contain a variety of different structural and regulatory components. Furthermore, the emerging views have pointed towards the proposition that each particle may play different biological roles depending upon various physiological contexts (Fig. 6). It would, therefore, be of critical significance to decipher the underlying mechanisms leading to these structural and functional diversities in centriolar satellite particles. Research of centriolar satellites will be even more prosperous over the next decade, and we will learn how they are organised in the context of cell cycle progression, environmental conditions and developmental programmes, why mutations in components and regulators of centriolar satellites cause human diseases and which strategies could be implemented to cure these disorders.

## Box: Anchoring of the microtubule minus end to the centrosome (related to *Delivery of microtubule*-*anchoring factors to the centrosome*)

In many, albeit not all, animal and fungal cells, microtubules emanate from and remain tethered to the centrosome, and are thereby organised into radial arrays around the centrosome (the spindle pole body (SPB) is the fungi equivalent of the animal centrosome) during interphase and assemble into bipolar spindle and astral microtubules during mitosis [141]. This anchorage ensures microtubule-mediated cellular processes, such as cell polarisation, cell movement, spindle assembly/orientation and chromosome segregation. Despite its prevailed phenomenon and biological significance, the molecular mechanism underlying microtubule anchoring had mostly been enigmatic until recently [142]. However, since the conserved protein family collectively called the MSD1/SSX2IP family consisting of fission yeast Msd1, filamentous fungus (*Aspergillus nidulans*) TINA, chicken LCG, murine ADIP and fish/frog/human SSX2IP [22, 24, 88, 143–147] was shown to comprise critical microtubule-anchoring factors, the underlying mechanism has begun to be revealed. The role for MSD1/SSX2IP in microtubule anchoring was first identified in fission yeast, in which Msd1 is required for tethering the minus ends of mitotic spindle microtubules to the SPB [143].

In contrast to fission yeast, in which Msd1 plays only a mitotic role in spindle microtubule anchoring, human MSD1/SSX2IP is required for microtubule anchoring during both interphase and mitosis. Intriguingly, in human cells, MSD1/SSX2IP is localised besides the centrosome at centriolar satellites in a PCM1-, microtubule- and dynein-dependent manner [22, 24]. It should be noted that fungal genomes do not contain PCM1 homologues, and centriolar satellites do not exist in these organisms. This diversification may explain the occurrence of the interphase role of the family members only in vertebrates and its absence in fission yeast and filamentous fungi [143, 146].

Fission yeast Msd1 interacts with an SPB-localising, microtubule nucleator, the γ-TuC [130], and thereby ensures microtubule anchoring to the SPB. Subsequent analysis [148] has further revealed that Msd1 forms a ternary complex with another conserved protein, Wdr8 (also called WRAP73) [149–151], and the minus end-directed Pkl1/kinesin-14 motor [152]. Pkl1 is responsible for transporting this ternary complex along spindle microtubules towards the SPB, to which Msd1 together with Wdr8 tethers Pkl1 by interacting with the γ-TuC. Pkl1 at the SPB in turn generates an inward pulling force, which antagonises the outward pushing force produced by the plus end-directed Cut7/kinesin-5 motor. Coordinated balance between the two forces exerted by these two antagonistic motors with opposite directionalities underlies the molecular mechanism by which the minus ends of mitotic spindles remain tethered to the SPB [148].

Interestingly, the formation of a protein complex between MSD1/SSX2IP and WDR8/WRAP73 is ubiquitously conserved across eukaryotes [26, 27, 29, 153]. Furthermore, the proper localisation of MSD1/SSX2IP is dependent upon WDR8/WRAP73 in humans as in fission yeast and filamentous fungi [26, 27, 148, 153]. However, evolutionary diversification between animals and fungi is seen in the minus end-directed motors involved. While kinesin-14 Pkl1 is responsible for SPB targeting of fission yeast Msd1 [148], the dynein motor plays an analogous role in MSD1/SSX2IP motility from centriole satellites to the centrosome in humans [24]. Consistent with this, dynein was previously reported to be involved in microtubule anchoring to the centrosome [154–156]. Nonetheless, the involvement of kinesin-14 in MSD1/SSX2IP recruitment to the centrosome, particularly during mitosis, cannot be excluded at present. In this regard, it is worth noting that the human kinesin-14 HSET was recently shown to form a complex with centrosomal CDK5RAP2/CEP215 that interacts with the γ-TuC, thereby ensuring microtubule anchoring and spindle pole clustering to the mitotic centrosome [157]. It is of note that another recent report shows that NEDD1, a metazoan-specific γ-TuC-binding protein, is required to anchor microtubules to the centrosome in mouse keratinocytes [158].

## Abbreviations

CS: Centriolar satellites
γ-TuC: γ-Tubulin complex
HU: Hydroxyurea
IR: Ionising radiation
MCPH: Microcephaly
MT: Microtubule
MTOC: Microtubule-organising centre
PCM: Pericentriolar material
SPB: Spindle pole body
UV: Ultraviolet
